# Effects of dietary tryptophan on the antioxidant capacity and immune response associated with TOR and TLRs/MyD88/NF-κB signaling pathways in northern snakehead, *Channa argus* (Cantor, 1842)

**DOI:** 10.3389/fimmu.2023.1149151

**Published:** 2023-04-11

**Authors:** Xin Zhang, Anran Wang, Enhui Chang, Bei Han, Jie Xu, Yu Fu, Xiaojing Dong, Shuyan Miao

**Affiliations:** Aquaculture Nutrition and Feed Laboratory, College of Animal Science and Technology, Yangzhou University, Yangzhou, China

**Keywords:** tryptophan, antioxidant capacity, TOR signaling pathway, TLRs/MyD88/NF-κB signaling pathway, northern snakehead *Channa argus*

## Abstract

**Introduction:**

Dietary tryptophan (Trp) has been shown to influence fish feed intake, growth, immunity and inflammatory responses. The purpose of this study was to investigate the effect and mechanism of Trp on immune system of juvenile northern snakehead (*Channa argus* Cantor, 1842).

**Methods:**

A total of 540 fish (10.21 ± 0.11 g) were fed six experimental diets containing graded levels of Trp at 1.9, 3.0, 3.9, 4.8, 5.9 and 6.8 g/kg diet for 70 days, respectively.

**Results and Discussion:**

The results showed that supplementation of 1.9-4.8 g/kg Trp in diets had no effect on the hepatosomatic index (HSI) and renal index (RI), while dietary 3.9 and 4.8 g/kg Trp significantly increased spleen index (SI) of fish. Dietary 3.9, 4.8, 5.9 and 6.8 g/kg Trp enhanced the total hemocyte count (THC), the activities of total antioxidant capacity (T-AOC) and superoxide dismutase (SOD). Malondinaldehyde (MDA) levels in the blood were significantly decreased by consuming 3.9 and 4.8 g/kg Trp. Fish fed with 3.0 and 3.9 g/kg Trp diets up-regulated interleukin 6 (*il-6*) and interleukin 8 (*il-8*) mRNA levels. The expression of tumor necrosis factor α (*tnf-α*) was highest in fish fed with 3.0 g/kg Trp diet, and the expression of interleukin 1β (*il-1β*) was highest in fish fed with 3.9 g/kg Trp diet. Dietary 4.8, 5.9 and 6.8 g/kg Trp significantly decreased *il-6* and *tnf-α* mRNA levels in the intestine. Moreover, Trp supplementation was also beneficial to the mRNA expression of interleukin 22 (*il-22*). Additionally, the mRNA expression levels of target of rapamycin (*tor*), toll-like receptor-2 (*tlr2*), toll-like receptor-4 (*tlr4*), toll-like receptor-5 (*tlr5*) and myeloid differentiation primary response 88 (*myd88*) of intestine were significantly up-regulated in fish fed 1.9, 3.0 and 3.9 g/kg Trp diets, and down-regulated in fish fed 4.8, 5.9 and 6.8 g/kg Trp diets. Dietary 4.8 and 5.9 g/kg Trp significantly increased the expression of inhibitor of nuclear factor kappa B kinase beta subunit (*ikkβ*) and decreased the expression of inhibitor of kappa B (*iκbα*), but inhibited nuclear transcription factor kappa B (*nf-κb*) mRNA level. Collectively, these results indicated that dietary 4.8 g/kg Trp could improve antioxidant capacity and alleviate intestinal inflammation associated with TOR and TLRs/MyD88/NF-κB signaling pathways.

## 1 Introduction

Amino acids play a significant role in regulating growth, immunity and intestinal health of animals (1, 2). As the lowest concentration of essential amino acids in most common protein sources (3), dietary tryptophan (Trp) has been found to influence the feed intake, growth, immunity and inflammatory responses of fish (4–12), while the regulatory mechanisms need to be further studied.

When fish are subjected to oxidative stress, the content of reactive oxygen species (ROS) in fish increases (13, 14), which can attack macromolecules such as proteins and nucleic acids in the organism, leading to oxidative damage (15). In addition, the lipid peroxidation of ROS with polyunsaturated fatty acids (PUFA) in the cell membrane, and the end products of peroxidation, such as malondialdehyde (MDA), have toxic effects on cells (16, 17). Total antioxidant capacity (T-AOC), superoxide dismutase (SOD) and catalase (CAT) are important components of the antioxidant defense system in fish, which can remove excess ROS in the body, and maintain normal physiological and life activities (16, 18). Supplementation of amino acids in the diet has improved antioxidant capacity and reduced oxidative stress in several fish (19–21). Study has been demonstrated that dietary Trp improved the activity of plasma antioxidant enzymes in juvenile blunt snout bream (*Megalobrama amblycephala*) (22). Dietary Trp has been reported to prevent the increase of MDA content in grass carp (*Ctenopharyngodon idella*) (23). However, the effect of dietary Trp on the enzymatic and nonenzymatic antioxidant capacity of northern snakehead, *Channa argus* (Cantor, 1842) remains to be investigated.

Intestine performs multiple functions, including digestion and absorption of nutrients, recognition of external factors, and signal transduction related to innate and adaptive immunity (24). As is well-known, intestinal cytokines are closely related to intestinal health (25). According to the research, the production of pro-inflammatory cytokines, such as interleukin 1 (IL-1), interleukin 6 (IL-6), and tumor necrosis factor-α (TNF-α), play a significant role in the development of intestinal inflammation (26). Accumulating evidence indicates that amino acids have powerful regulatory roles in cell signaling and mRNA translation (27, 28). Trp was reported to exert beneficial regulatory function in mucosal growth or maintenance, as well as alleviation of intestinal inflammation by 5-hydroxytryptophan (5-HT) (29). Target of rapamycin (TOR) and nuclear factor-kappa B (NF-κB) signaling pathways have been considered to have momentous functions in cell proliferation, differentiation, growth, and metabolism (30, 31). In addition, a large number of studies have shown that various amino acids can regulate intestinal inflammation through the TOR or NF-κB signaling pathway (32–34). Glutamine was found to attenuate intestinal inflammation dependent on its function *via* the mechanistic target of rapamycin (mTOR) and NF-κB signaling pathways (35, 36). Studies have shown that when the TLRs/MyD88/NF-κB signal pathway is inhibited, the inflammatory response in *Oreochromis niloticus* decreases (37). However, there are few reports on the effects and mechanisms of dietary Trp on immunoregulation of *Channa argus*, which need to be further investigated.

*C. argus* is one of the main economic species in China, due to its rich edible and medicinal value (38). The production of *C. argus* in 2021 was about 548,500 tons (39). To our knowledge, limited information about the nutritional immunity of *C. argus* is available (40, 41). In our previous research, based on the second-degree polynomial regression analysis of specific growth rate and feed efficiency against dietary Trp levels, the optimum dietary Trp requirements for *C. argus* were respectively estimated to be 4.6 and 4.5 g/kg (42). Therefore, in the present study, we investigated the effects of different dietary Trp levels on the antioxidant capacity and intestinal health of *C. argus*, and discussed its possible mechanisms of immune regulation by analyzing the expressions of related genes of the signaling pathways.

## 2 Materials and methods

### 2.1 Experimental diets

The formulation and proximate composition of the basal diet are shown in **Table 1**. Fish meal, poultry by-product meal and mixed L-amino acids were chosen as the main protein sources, fish oil and soybean oil were chosen as the main lipid sources. 1.0, 2.0, 3.0, 4.0 and 5.0 g/kg diet Trp (Sinopharm Chemical Reagent Co., Ltd, SHH, CHN) were added into the basal diet to produce five experimental diets, respectively. Trp supplement is balanced with glutamate, accounting for 1% of the diet. Prior to the addition of oil and water, all raw ingredients were ground through a 246-micron sieve, then were fully mixed and extruded into particles with diameter of 2.0 × 3.0 mm. Finally, all diets were dried at 40°C and stored at -20°C until use.

**Table 1:** Formulation and proximate analysis of the basal diet (g/kg diet).

Ingredient	Content
Fish meal (70.2% crude protein)	105.00
Poultry by-product meal (66.5% crude protein)	200.00
Wheat meal (12.0% crude protein)	150.00
Gelatin (97.5% crude protein)	40.00
Yeast powder (58.2% crude protein)	30.00
Wheat bran (18.6% crude protein)	110.00
Fish oil	25.00
Soybean oil	30.00
Vitamin mixture ^a^	15.00
Mineral mixture ^b^	15.00
Amino acid mixture ^c^	150.00
Calcium dihydrogen phosphate	10.00
Choline chloride	10.00
Cellulose	99.00
Dimethyl-β-propiothetin	1.00
Composition (% dry weight basis)	
Moisture	7.00
Crude protein	48.56
Crude lipid	10.38
Crude ash	10.04
Carbohydrate^1^	24.02
Gross energy^2^, kJ/g	19.72

a.Vitamin premix (mg/kg diet): vitamin B_1_, 25 mg; vitamin B_2_, 45 mg; vitamin B_12_, 10 mg; vitamin B_6_, 20 mg; vitamin K, 10 mg; vitamin A, 32 mg; vitamin C, 2000 mg; vitamin D_3_, 5 mg; vitamin E, 240 mg; niacin acid, 200 mg; folic acid, 20 mg; biotin, 60 mg; calcium pantothenate, 60 mg; micocrystalline cellulose 12273 mg.

b Mineral premix (mg/kg diet): inositol, 800 mg; MgSO_4_·H_2_O, 1200 mg; CuSO_4_·5H_2_O, 10 mg; FeSO_4_·H_2_O, 80 mg; ZnSO_4_·H_2_O, 50 mg; MnSO_4_·H_2_O, 45 mg; CoCl_2_·6H_2_O, 50 mg; Ca(IO_3_)_2_, 60 mg; Na_2_SeO_3_, 20 mg; zeolite powder, 12685 mg.

c Amino acid mixture (g/kg diet): arginine, 9.98 g; histidine, 3.37 g; isoleucine, 9.91 g; leucine, 18.60 g; lysine, 23.52 g; methionine, 6.27 g; cysteine, 10.27 g; phenylalanine, 7.57 g; threonine, 10.79 g; valine, 8.48 g; aspartic acid, 28 g; glycine, 14 g.

1 Carbohydrate (%) = 100 - (% crude protein + % crude lipid + % moisture + % ash).

2 Calculated based on 17.2 kJ g^−1^ carbohydrate; 23.6 kJ g^−1^ protein and 39.5 kJ g^−1^ lipid according to the method described in a previous study (43).

Amino acid contents of diets were analyzed with automatic amino acid analyzer (L-8900, Hitachi, Japan). The amino acid profile of the basal diet is presented in **Table 2**. The final Trp content in six diets are 1.9, 3.0, 3.9, 4.8, 5.9 and 6.8 g/kg, respectively.

**Table 2:** The amino acid profile of the basal diet (% dry weight).

Amino acid	Amino acid composition of basal diet	
Essential amino acids (EAAs)		
Lysine	4.29	
Methionine	0.99	
Arginine	2.89	
Phenylalanine	2.18	
Histidine	1.01	
Isoleucine	2.04	
Leucine	3.93	
Threonine	2.21	
Valine	2.10	
Tryptophan	0.19	
Non-essential amino acids (NEAAs)		
Aspartic acid	5.06	
Glutamic acid	4.65	
Serine	1.22	
Glycine	4.15	
Alanine	2.01	
Tyrosine	0.87	
Cysteine	1.10	

### 2.2 Fish and culturing conditions

Healthy *C. argus* were purchased from a commercial hatchery in Guangdong, China. Before the trial, fish were acclimated to experimental conditions in the greenhouse in Yangzhou University with a water-recirculating system, and fed with the basal diet for two weeks. Then, 540 fish with initial weight of 10.21 ± 0.11 g were randomly assigned to 18 cages (1 m × 1 m × 80 cm) with 30 fish in each cage, and three cages of fish were randomly provided for each experimental diet. During the feeding trial, all fish were fed at 8:00 and 17:00 daily at apparent satiation level. Feed consumption and fish mortality were recorded every day. The water dissolved oxygen was 6.2-6.6 mg/L and the temperature was 25.5-28.5°C. All experimental protocols were approved by the Animal Care Advisory Committee of Yangzhou University.

### 2.3 Sample collection and analysis

After 70 days feeding trial, all *C. argus* were fasted for 24 h, then anaesthetized by MS-222 solution (250 mg/L, Sigma). Liver, head kidney and spleen samples were collected from 10 fish in each cage and weighed to calculate the hepatosomatic index (HSI), renal index (RI) and spleen index (SI). Blood samples were collected in heparin sodium-anticoagulant tubes from 10 fish in each cage and divided into two parts, one part was prepared for the determination of malondinaldehyde (MDA) value and the antioxidant enzymes activity, including superoxide dismutase (SOD), catalase (CAT) and total antioxidant capacity (T-AOC), another part was prepared for the determination of total hemocyte count (THC). The whole intestine samples were collected from 5 fish in each cage for determination of the relative mRNA levels of genes, including tumor necrosis factor α (*tnf-α*), interleukin 1β (*il-1β*), interleukin 6 (*il-6*), interleukin 8 (*il-8*), interleukin 22 (*il-22*), target of rapamycin (*tor*), toll-like receptor-2 (*tlr2*), toll-like receptor-4 (*tlr4*), toll-like receptor-5 (*tlr5*), myeloid differentiation factor 88 (*myd88*), inhibitor of nuclear factor kappa B kinase beta subunit (*ikkβ*), inhibitor of kappa B (*iκbα*), nuclear transcription factor kappa B (*nf-κb*).

The proximate composition of the ingredients and diet, including the moisture, crude protein, crude lipid and crude ash were determined using standard procedures of the AOAC (44). The content of amino acids in ingredients and diet was determined by the method of Rajendra (45). THC was measured and calculated according to Sierra et al. (46). MDA value and the antioxidant enzymes activity were measured using commercial kits provided by Jian Cheng Bioengineering Institute, Nanjing, China. The relative mRNA level of genes was analyzed by RT-*q*PCR method according to Miao et al. (47). The cDNA sequences of the relevant genes were queried in the NCBI, and all primers were designed using Primer Premier 6. All specific primers for genes are provided in **Table 3**. *β-actin* was chosen as the internal reference gene based on the preliminary tests and Norm Finder algorithms (48, 49).

**Table 3:** Primers sequence for RT-*q*PCR.

Gene	Forward primer (5’-3’)	Reverse primer (5′–3′)
*β-actin*	CACTGTGCCCATCTACGAG	CCATCTCCTGCTCGAAGTC
*tor*	GAGCCTCTCTCATCCTCACCAC	GATTCATTCCTTTCTCTTTAGCCA
*tlr-2*	CTGGACGAATCATCGAATCACCT	AACTTTGGCTTCCTCTTGGCTCT
*tlr-4*	GGAGGAGACAGAAGGTGTAGATTTG	AGGTTGTGATCTTGGGCTGAGTG
*tlr-5*	ACCTCTTCCGCTGTTGTTTCG	AGTGAGCCACCTTCCCTACCA
*myd88*	TGTCCGAGGTGGAAAGAAGTG	TCAAAGTCGCTCTGGCAGTAG
*ikkβ*	ATCACAGAGCAACCCCTTTT	CCACTGTAGTTAGGGAAGGA
*iκbα*	AAAATGTTACCGTGCCAGGAC	ATGTATCACCGTCGTCAGTC
*nf-κb*	CAGCCAAAACCAAGAGGGAT	TCGGCTTCGTAGTAGCCATG
*tnf-α*	ACAATACCACCCCAGGTCCCA	ACGCAGCATCCTCTCATCCAT
*il-1β*	ATGACATGCAATGTGAGCAAAAT	TTAACTCGTATGCTGAATGGTGA
*il-6*	CATGGAGCACTCAAAGAGGATAG	CTGAGGTGGAGGTAGTGTTGTCG
*il-8*	GAGTCTGAGCAGCCTGGGAGT	CTGTTCGCCGGTTTTCAGTG
*il-22*	CAGGCTGTGCAGACGGAGGAAGA	GCGTGGTGATGGTCGTGATAGTGAG

### 2.4 Calculations and statistical analysis

Hepatosomatic index (HSI), spleen index (SI), and renal index (RI) were calculated as following:


$$HSI\;\left(\%\right)=100\times \frac{w_{h}}{w_{b}}$$

$$SI\;\left(\%\right)=100\times \frac{w_{s}}{w_{b}}$$

$$RI\;\left(\%\right)=100\times \frac{w_{r}}{w_{b}}$$

W_b_: fish weight (g); W_h_: liver weight (g); W_s_: spleen weight (g); W_r_: head kidney weight (g)

After homogeneity variance tests by SPSS 18.0 (SPSS Inc., Chicago, IL, USA), the data (means ± S.D.) were subjected to a one-way analysis of variance (ANOVA) by Tukey’s multiple comparison test to assess the significant differences among the treatments at *P* < 0.05. In addition, to determine if the effect was linear and/or quadratic, a follow-up trend analysis using orthogonal polynomial contrasts was performed (50).

## 3 Results

### 3.1 Organ index

**Figure 1:**
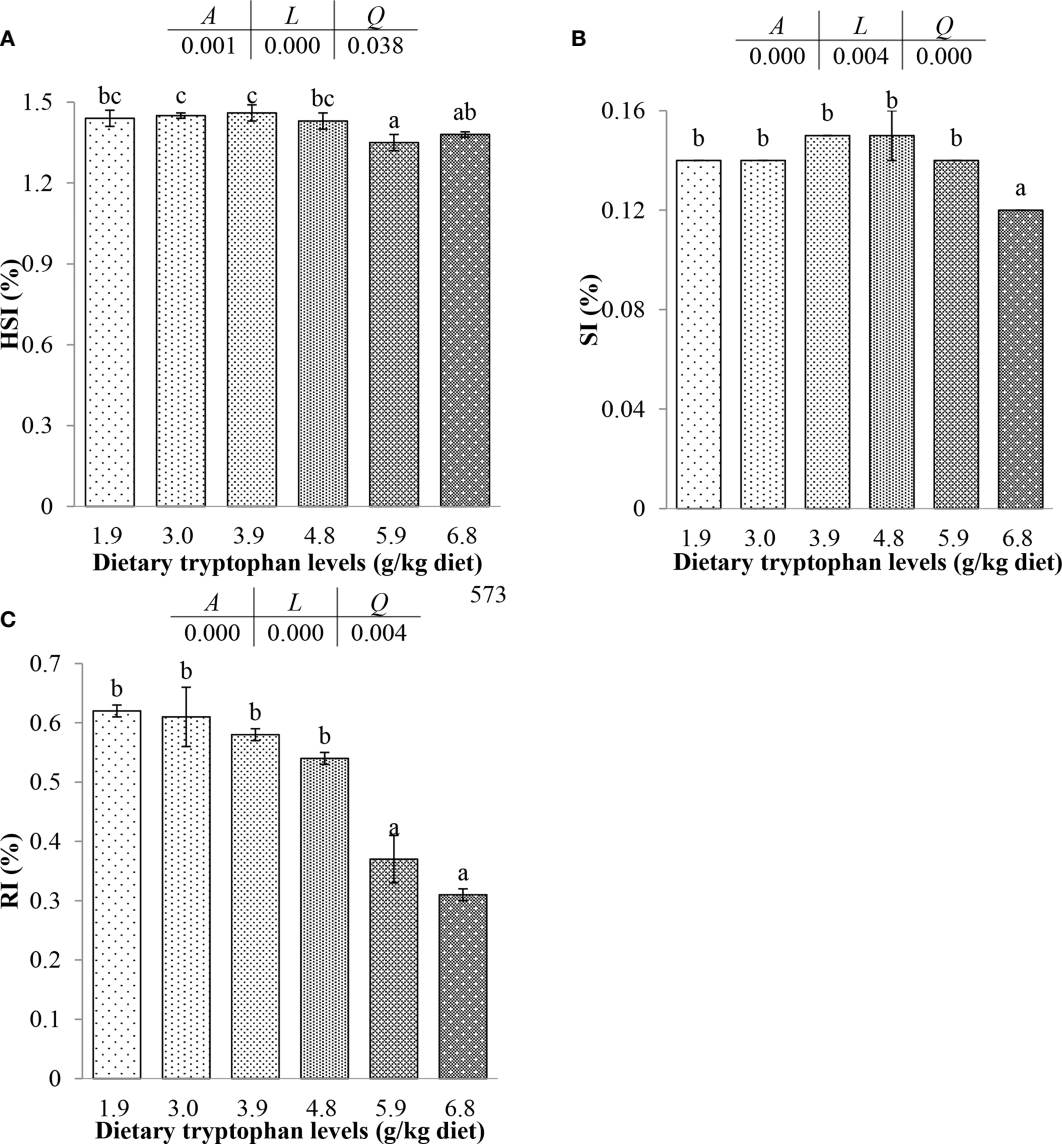
Effects of dietary tryptophan with different levels on organ index of *C. argus* (means ± S.D. of three replications). Bars with different letters indicate significantly among the treatments (*P* < 0.05). **(A)** Hepatosomatic index (HSI); **(B)** Spleen index (SI); **(C)** Renal index (RI).

The effects of dietary Trp on organ index and THC of *C. argus* are shown in **Figure 1**. The HSI and RI in fish fed the 1.9, 3.0, 3.9 and 4.8 g/kg Trp diets were significantly higher than that in fish fed the 5.9 g/kg Trp diet (*P* < 0.05), and there was no significant difference among fish fed the 1.9, 3.0, 3.9 and 4.8 g/kg Trp diets (*P* > 0.05). The SI and HSI of fish increased at first and then decreased as Trp content increased. Fish fed 3.9 and 4.8 g/kg Trp diets had the highest SI. There were significantly negative linear and positive quadratic trends between the dietary Trp levels and the dependent variables including HSI, SI and RI (*P* < 0.05).

### 3.2 Total hemocyte count and hematologic antioxidant-related parameters

**Figure 2:**
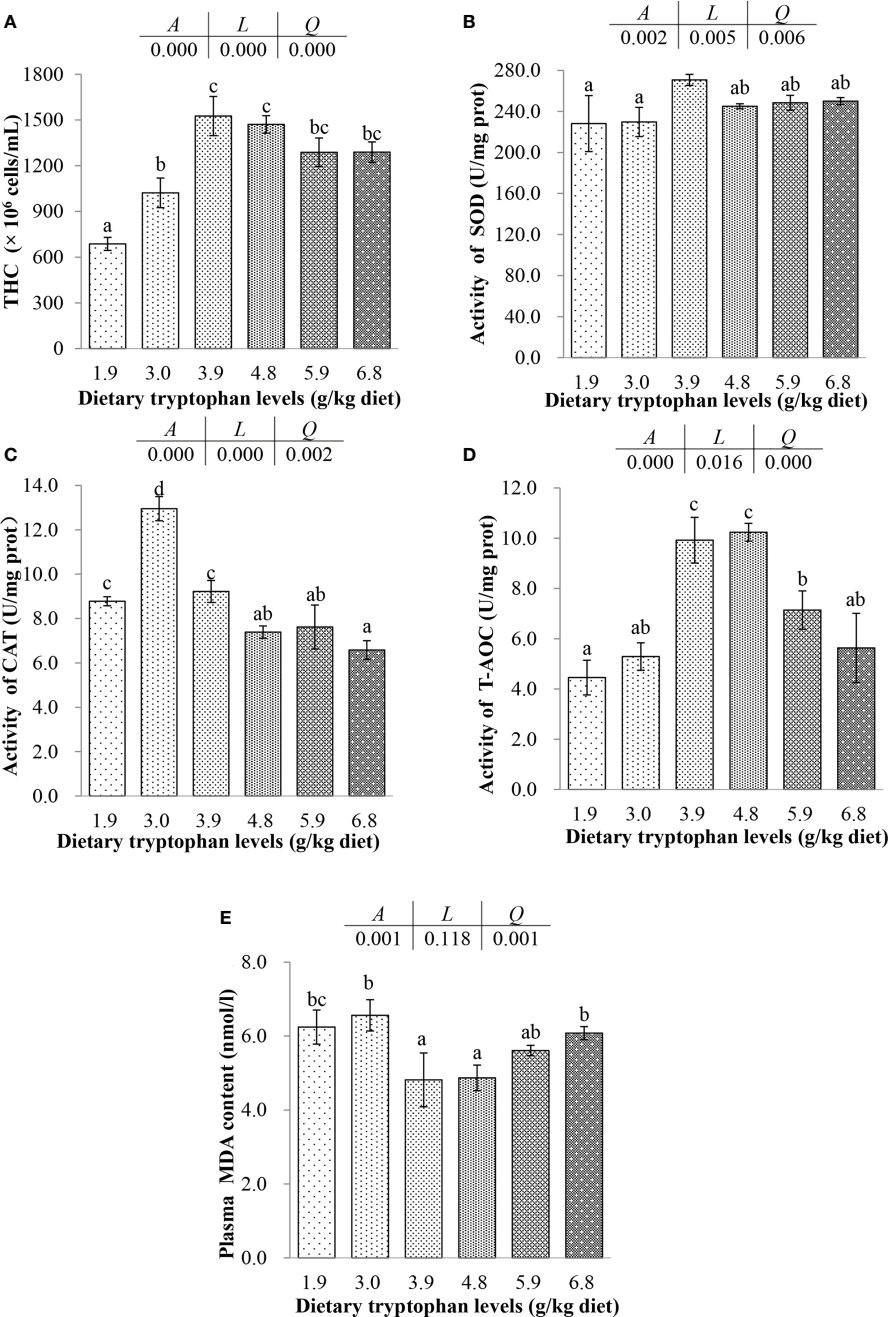
Effects of dietary tryptophan different levels on total hemocyte count and hematologic antioxidant parameters of blood in *C argus* (means ± S.D. of three replications). Bars with different letters differ significantly among the treatments (*P* < 0.05). **(A)** Total hemocyte count (THC); **(B)** Superoxide dismutase (SOD); **(C)** Catalase (CAT); **(D)** Total antioxidant capacity (T-AOC); **(E)** Malondialdehyde (MDA).

The effects of dietary Trp level on THC and the hematologic antioxidant-related parameters are shown in **Figure 2**. The THC levels in *C. argus* increased as Trp levels increased until the dietary Trp level reached 3.9 g/kg, then began to decrease when the dietary Trp level exceeded 4.8 g/kg, and had significantly positive linear and negative quadratic trends with dietary Trp levels (*P* < 0.05).

With the increase of Trp, the activities of SOD, CAT and T-AOC were increased first and then decreased. SOD reached the highest activity in fish fed the diet with 3.9 g/kg Trp, and T-AOC reached the maximum activity in fish fed the 3.9 and 4.8 g/kg Trp diets (*P* < 0.05). However, the highest CAT activity was showed in fish fed the diet with 3.0 g/kg Trp (*P* < 0.05), but there was no significant difference between the fish fed the 1.9 and 3.9 g/kg Trp diets (*P* > 0.05), and the activities of CAT in fish fed the 1.9-3.9 g/kg Trp diets were significantly higher than that in other fish (*P* < 0.05). There were significantly linear and positive quadratic trends between the dietary Trp levels and the dependent variables including SOD, T-AOC and CAT (*P* < 0.05). However, the MDA contents had positive quadratic trend with dietary Trp levels (*P* < 0.05). The MDA contents in fish fed the 3.9 and 4.8 g/kg Trp diets were significantly decreased compared with those in fish fed the 1.9, 3.0 and 6.8 g/kg Trp diets (*P* < 0.05), and there was no significant difference with fish fed the 5.9 g/kg Trp diet (*P* > 0.05).

### 3.3 Relative mRNA expression of genes related to intestinal inflammatory factors

**Figure 3:**
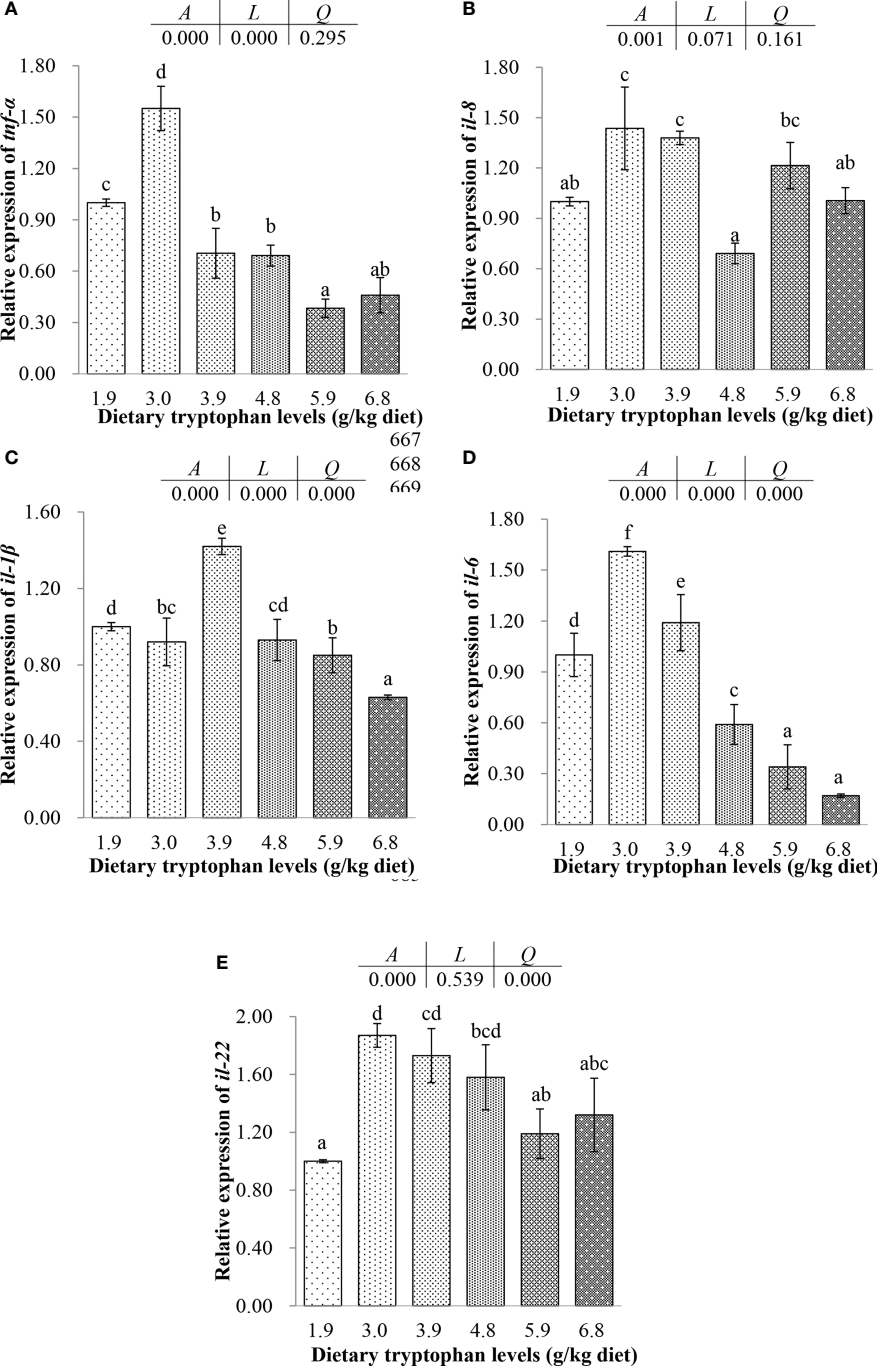
Effects of dietary tryptophan with different levels on the relative mRNA expression of genes related to intestinal inflammatory factors of *C argus* (means ± S.D. of three replications). Bars with different letters differ significantly among the treatments (*P* < 0.05). **(A)** Tumor necrosis factor α (*tnf-α*); **(B)** Interleukin 8 (*il-8*); **(C)** Interleukin 1β (*il-1β*); **(D)** Interleukin 6 (*il-6*); **(E)** Interleukin 22 (*il-22*).

As shown in **Figure 3**, dietary Trp level significantly affected the relative expressions of genes related to intestinal inflammatory factors, including interleukins and TNF-α (*P* < 0.05).

The expressions of *tnf-α*, *il-6* and *il-8* increased first and then decreased with dietary 1.9-4.8 g/kg Trp. There were significantly negative linear trends between the dietary Trp levels and the dependent variables including *tnf-α* and *il-6* (*P* < 0.05). For *il-6* and *il-8*, the expressions of them in fish fed the 4.8 g/kg Trp diet were significantly lower than those fed with 1.9, 3.0 and 3.9 g/kg Trp diets (*P* < 0.05), and the expressions of them were higher in fish fed the 3.0-3.9 g/kg Trp diets compared with 1.9 g/kg Trp diet group (*P* < 0.05). The expression of *tnf-α* was highest in fish fed 3.0 g/kg Trp diet and significantly decreased in fish fed 3.9 and 4.8 g/kg Trp diets compared to fish fed the basal diet (*P* < 0.05). The expression of *il-1β* in fish fed diets with 3.0, 4.8, 5.9 and 6.8 g/kg Trp showed a decreasing trend compared with the control group, but there was no significant difference except for dietary 6.8 g/kg Trp (*P* > 0.05). There were significantly negative quadratic trends between the dietary Trp levels and the dependent variables including *il-1β*, *il-6* and *il-22* (*P* < 0.05). The higher expression of *il-22* was found in fish fed diets with 3.0-4.8 g/kg Trp (*P* < 0.05), and there was no significant difference between the fish fed diets with 3.0-4.8 g/kg Trp (*P* > 0.05).

### 3.4 Relative mRNA expression of genes related to the intestinal target of signaling pathways

**Figure 4:**
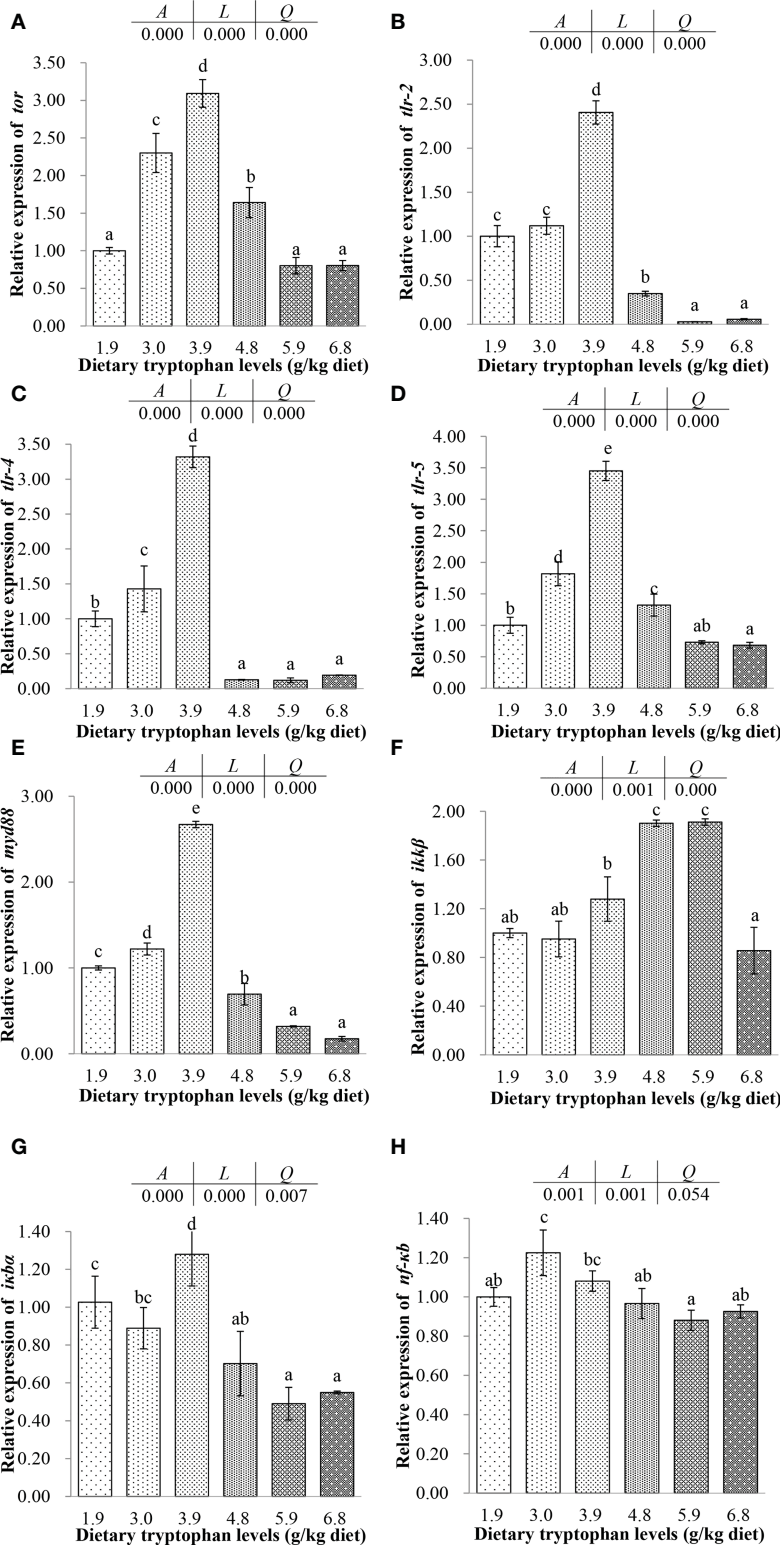
Effects of dietary tryptophan with different levels on the relative mRNA expression of genes related to the TOR and NF-κB signaling pathways of *C argus* intestine (means ± S.D. of three replications). Bars with different letters differ significantly among the treatments (*P* < 0.05). **(A)** Target of rapamycin (*tor*); **(B)** Toll-like receptor-2 (*tlr-2*); **(C)** Toll-like receptor-4 (*tlr4*); **(D)** Toll-like receptor-5 (*tlr5*); **(E)** Myeloid differentiation factor88 (*myd88*); **(F)** Inhibitor of nuclear factor kappa B kinase beta subunit (*ikkβ*); **(G)** Inhibitor of kappa B (*iκbα*); **(H)** Nuclear factor-κ-gene binding (*nf-κb*).

As shown in **Figure 4**, there were significantly linear and positive quadratic trends between the dietary Trp levels and the expressions of *tor*, *tlr-2*, *tlr-4*, *tlr-5*, *myd88*, *iκbα* and *ikkβ* (*P* < 0.05).

The expressions of *tor*, *tlr-2*, *tlr-4*, *tlr-5* and *myd88* were all significantly increased with the dietary Trp increasing from 1.9 to 3.9 g/kg, and then decreased (*P* < 0.05). The expressions of *tlr-2*, *tlr-5*, *myd88* and *iκbα* in fish fed the 4.8 g/kg Trp diet were significantly lower than those in fish fed the basal diet (*P* < 0.05). Moreover, the lowest *tor*, *tlr-2*, *tlr-5* and *myd88* gene expressions were found in fish fed the 5.9 and 6.8 g/kg Trp diets (*P* < 0.05). The expression of *ikkβ* increased with increasing dietary Trp up to 4.8 g/kg (*P* < 0.05) and decreased with increasing dietary Trp up to 6.8 g/kg (*P* < 0.05). The expression of *iκbα* showed a positive linear except for that in the fish fed diet with the 3.9 g/kg Trp. At the same time, the expressions of *iκbα* were lower in the fish fed with 4.8, 5.9 and 6.8 g/kg Trp diets (*P* < 0.05), compared with the fish fed with basal diet. The expressions of *nf-κb* in the fish fed with 4.8, 5.9 and 6.8 g/kg Trp diets were lower than those in fish fed the basal diet, but there was no significant difference (*P* > 0.05). The expression of *nf-κb* was highest in the fish fed with 3.0 g/kg Trp diet (*P* < 0.05), and had significantly positive linear trend with dietary Trp levels (*P* < 0.05).

## 4 Discussion

The organ index usually reflects the development of organs and the general nutritional status of animals (51). The liver, spleen and kidney are the main immune organs of fish, which can reflect the immune state of fish to some extent (52). In the present study, HSI, SI and RI decreased significantly in linear and quadratic curves with dietary Trp increasing, but there was no significant difference in fish fed with 1.9-4.8 g/kg Trp diets. Sharf et al. (53) showed that HSI and viscera somatic index of fingerling *Channa punctatus* decreased with the increase of dietary 0.9-9.1 g/kg Trp. However, studies have shown that dietary supplemented with 0.4-0.6 g/kg Trp could significantly increase SI of ducks (54). Carrillo-Vico et al. (55) also proved that melatonin (the metabolic product of Trp) could positively stimulate the development of spleen. In the present study, although dietary 3.9 or 4.8 g/kg Trp couldn’t significantly improve the development of spleen, it has shown a significantly negative quadratic trend, which may be due to the differences in culturing time and breeding subjects. These results provide a hint that the optimum supplementation of Trp in feed was between 3.9 and 4.8 g/kg, which may be beneficial to the development of spleen and had no negative effect on the liver and head-kidney.

The function of blood is closely related to maintaining the stability of various physiological environments in the fish, such as eliminating invading bacteria, phagocytosis of foreign body particles and participating in immune response (56, 57). In this study, the THC in *C. argus* increased at first and then decreased with the increase in dietary Trp level, and there was a significant linear and quadratic relationship. Studies have shown that changes of THC are related to the health status of spleen, liver and other hematopoietic organs (58). This phenomenon was also observed in this study, when inclusion of 3.9-4.8 g/kg Trp in the diets, the SI of *C. argus* showed an upward trend, accompanied by the highest THC. In aquatic animals, antioxidant systems served as the first line of defense against oxidative damage (59). SOD, CAT, T-AOC are commonly used to evaluate the antioxidant capacity and immune response of aquatic animals (11, 32, 59, 60). The present study showed that the activities of SOD and T-AOC in blood were significantly increased when fish fed with 3.9-4.8 g/kg Trp. These results are similar to the findings in the liver of pigs (61), intestine of young grass carp (32). Meanwhile, MDA levels in tissues can be used to estimate lipid peroxidation (62). In this study, the MDA contents in the blood of *C. argus* were significantly decreased with dietary 3.9-4.8 g/kg Trp. Similar results also have been observed in hybrid catfish (*Pelteobagrus vachelli*♀ ×*Leiocassis longirostris*♂) (11). In this study, the activity of CAT in blood was highest in fish feed with 3.0 g/kg Trp diet. However, dietary 4.0 g/kg Trp significantly increased the activity of CAT in juvenile blunt snout bream (22). The reason may be caused by different kinds of fish. These findings suggested that dietary 3.9 and 4.8 g/kg Trp can enhance the antioxidant capacity of *C. argus* by increasing of SOD and T-AOC activities and decreasing the contents of MDA in the blood.

Intestinal cytokines, such as interleukins (ILs) and tumor necrosis factors (TNFs), are important components of the fish mucosal immune system (63). Pro-inflammatory cytokines including TNF-α, IL-1β, IL-6 and IL-8 can promote the occurrence of inflammatory reactions (64, 65), and anti-inflammatory cytokines such as IL-22 can promote host immune defense against bacterial pathogens (66). In the present study, when dietary Trp reached 4.8 g/kg, the relative expressions of *tnf-α*, *il-1β*, *il-6* and *il-8* in intestine of *C. argus* decreased, whereas the relative expression of *il-22* increased significantly. Trp have been shown to have a similar effect in other study (67). The TOR signaling pathway plays a critical role in the immune system of monocytes (68). And the TOR signaling pathway may improve the innate immune system of fish and human by regulating the transcription of cytokines (69, 70). In the present study, we observed that the expressions of *tor*, *il-1β*, *il-6* and *il-8* were significantly increased when the dietary Trp was up to 3.9 g/kg, but the opposite results were observed when dietary Trp reached 4.8 g/kg. Meanwhile, recent studies have shown that dietary Trp may up-regulate anti-inflammatory factors and down-regulate pro-inflammatory factors of fish partly by regulating the transcription of TOR (5, 22). These results demonstrated that the optimum dietary Trp level could alleviate intestinal inflammation partly by down-regulating the expressions of *tnf-α*, *il-1β*, *il-6* and *il-8*, and up-regulating the expression of *il-22* in fish intestine *via* regulating the expression of *tor*. However, the underlying mechanism by which dietary Trp attenuates inflammatory responses through the TOR signaling pathway remains to be further studied.

Furthermore, the NF-κB translocates to the nucleus and upregulates the expression of genes linked with inflammation, cell survival, proliferation, invasion, and angiogenesis (71), and mediated the proinflammatory action of TOR (70). The increased expression of IKK complex (including IKKα, IKKβ and IKKγ) promotes the phosphorylation and degradation of IκBα, which in turn activates NF-κB, which suppresses the relative expression of anti-inflammatory cytokines and up-regulates the relative expression of pro-inflammatory cytokines (72). In the present study, the relative expression of *ikkβ* increased and the expression of *iκbα* decreased significantly with dietary Trp up to 5.9 g/kg, but there was no difference in *nf-κb*. These immune responses may be caused by multiple pathways regulating NF-κB (73), such as TLRs/MyD88/NF-κB signaling pathway. TLRs (including TLR2, TLR4 and TLR5) are transmembrane proteins that can recognize a variety of related molecules (such as lipopolysaccharide, sodium urate crystal, viral double-stranded RNA, etc.) and cause inflammatory immune response. TLRs can activate the MyD88-dependent pathways, thus activating NF-κB and resulting in the release of inflammatory mediators and cytokines (74, 75). In the present study, the relative expressions of *tlr2* and *tlr4* showed a certain positive correlation with that of *myd88* when dietary Trp 1.9-4.8 g/kg. At the same time, dietary 4.8 g/kg Trp inhibited the relative expression of *nf-κb*, which is consistent with the relative expressions of *tnf-α*, *il-1β*, *il-6* and *il-8*. Li et al. (52) also observed similar results in the kidney of juvenile blunt snout bream fed with 2.8 or 4.0 g/kg Trp. These results implied that the optimum dietary Trp may regulate inflammatory cytokines through the TLRs/MyD88/NF-κB signaling pathway.

**Figure 5:**
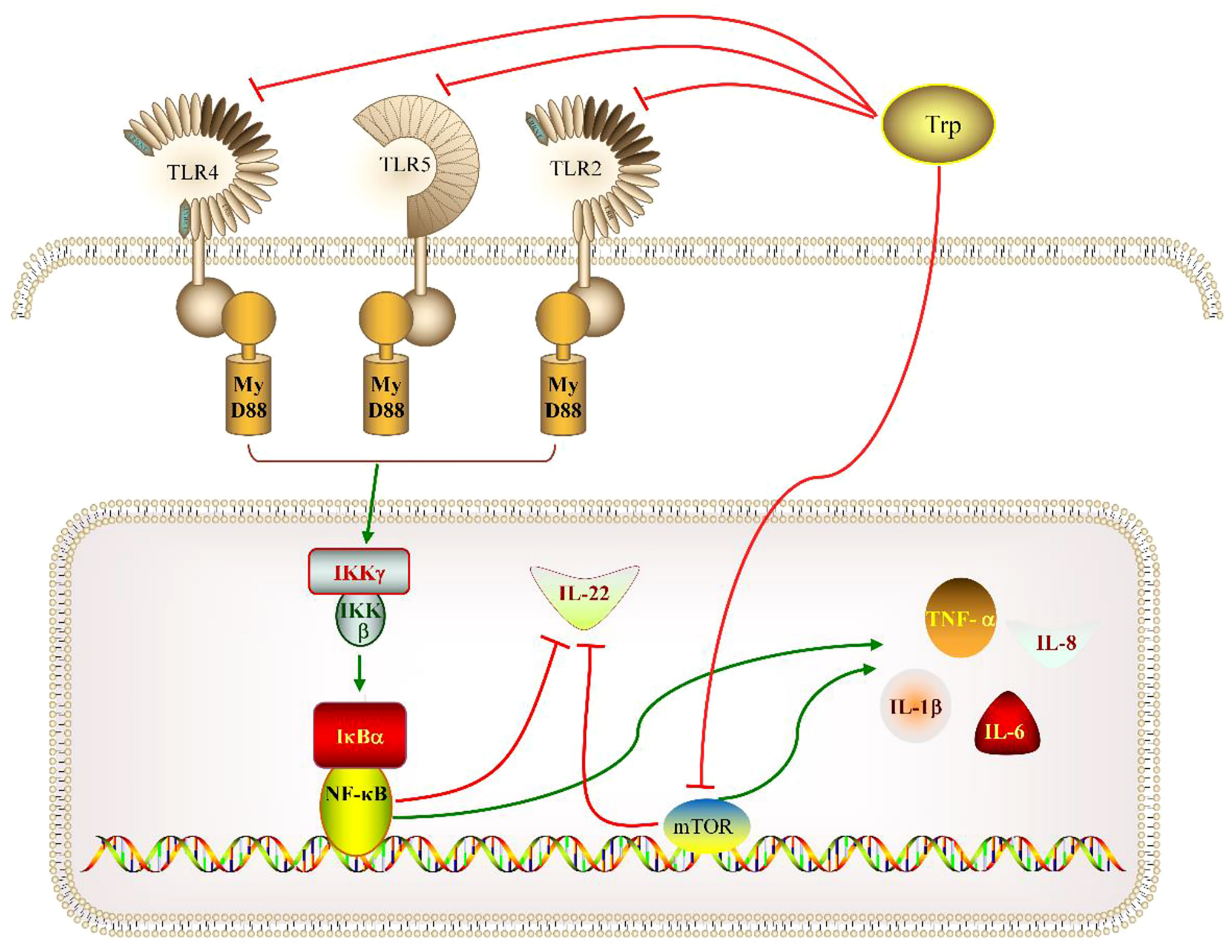
Possible mechanisms of the mechanisms of optimal dietary tryptophan promoted intestinal health *via* TOR and TLRs/MyD88/NF-κB signaling pathways.

In conclusion, the present study provided evidence that dietary 3.9-4.8 g/kg Trp could improve total hemocyte count, antioxidant enzyme activity in blood, and decrease the content of MDA in blood, but have no effect on the development of liver, spleen and head-kidney. Furthermore, dietary 4.8 g/kg Trp could alleviate intestinal inflammation partly by down-regulating the expression of *tnf-α*, *il-1β*, *il-6* and *il-8* and up-regulating the expression of *il-22* in fish intestine *via* the TOR and TLRs/MyD88/NF-κB signaling pathways (**Figure 5**).

## Data availability statement

The original contributions presented in the study are included in the article. Further inquiries can be directed to the corresponding author.

## Ethics statement

The animal study was reviewed and approved by Animal Care Advisory Committee of Yangzhou University.

## Author contributions

XZ: data curation and writing-original draft. AW: writing-reviewing and editing. EC: software. BH: investigation and formal analysis. JX and YF: formal analysis. XD: writing-reviewing and editing. SM: conceptualization, methodology and funding acquisition. All authors contributed to the article and approved the submitted version.

## References

[1] Wang WW Qiao SY Li DF. Amino acids and gut function. Amino Acids (2008) 37:105–10. doi: 10.1007/s00726-008-0152-4 18670730

[2] Li P Mai K Trushenski J Wu G. New developments in fish amino acid nutrition: Towards functional and environmentally oriented aquafeeds. Amino Acids (2008) 37:43–53. doi: 10.1007/s00726-008-0171-1 18751871

[3] Farhat Khan MA. Dietary l-tryptophan requirement of fingerling stinging catfish, *Heteropneustes fossilis* (Bloch). Aquac Res (2014) 45:1224–35. doi: 10.1111/are.12066

[4] Cabanillas-Gámez M Bardullas U Galaviz MA Rodriquez S Rodriquez VM López LM. Trp supplementation helps totoaba (*Totoaba macdonaldi*) juveniles to regain homeostasis in high-density culture conditions. Fish Physiol Biochem (2020) 46:597–611. doi: 10.1007/s10695-019-00734-2 31820206

[5] Tang L Feng L Sun CY Chen GF Jiang WD Hu K. Effect of tryptophan on growth, intestinal enzyme activities and TOR gene expression in juvenile jian carp (*Cyprinus carpio* var. jian): studies in vivo and *in vitro* . Aquaculture (2013) 412-413:23–33. doi: 10.1016/j.aquaculture.2013.07.002

[6] Ramos-Pinto L Martos-Sitch JA Reis B Azeredo R Fernandez-Boo S Pérez-Sánchez J. Dietary tryptophan supplementation induces a transient immune enhancement of gilthead seabream (*Sparus aurata*) juveniles fed fishmeal- free diets. Fish Shellfish Immun (2019) 93:240–50. doi: 10.1016/j.fsi.2019.07.033 31310850

[7] Richard DM Dawes MA Mathias CW Acheson A Hill-Kapturczak N Dougherty DM. L-trp: basic metabolic functions, behavioral research and therapeutic indications. Int J Tryptophan Res (2009) 2:45–60. doi: 10.4137/IJTR.S2129 20651948 PMC2908021

[8] Meyer-Gerspach AC Hafliger S Meili J Doody A Rehfeld JF Drewe J. Effect of l-trp and l-leucine on gut hormone secretion, appetite feelings and gastric emptying rates in lean and non-diabetic obese participants: A randomized, double-blind, parallel-group trial. PloS One (2016) 11(11):e0166758. doi: 10.1371/journal.pone.0166758 27875537 PMC5119776

[9] Steinert RE Luscombe-Marsh ND Little TJ Scott S Bärbel O Michael H. Effects of intraduodenal infusion of l-trp on ad libitum eating, antropyloroduodenal motility, glycemia, insulinemia, and gut peptide secretion in healthy men. J Clin Endocr Metab (2014) 99:3275–84. doi: 10.1210/jc.2014-1943 24926954

[10] Rothhammer V Mascanfroni ID Bunse L Takenaka MC Kenison JE Mayo L. Type I interferons and microbial metabolites of trp modulate astrocyte activity and central nervous system inflammation *via* the aryl hydrocarbon receptor. Nat Med (2016) 22:586–97. doi: 10.1038/nm.4106 PMC4899206 27158906

[11] Zhao Y Wu XY Xu SX Xie JY Xiang KW Feng L. Dietary tryptophan affects growth performance, digestive and absorptive enzyme activities, intestinal antioxidant capacity, and appetite and GH–IGF axis-related gene expression of hybrid catfish (*Pelteobagrus vachelli*♀ × *Leiocassis longirostris*♂). Fish Physiol Biochem (2019) 45:1627–47. doi: 10.1007/s10695-019-00651-4 31161532

[12] Melchior D Mézière N Sève B Le Floc’h N. Is tryptophan catabolism increased under indoleamine 2, 3 dioxygenase activity during chronic lung inflammation in pigs? Reprod Nutr Dev (2005) 45:175–83. doi: 10.1051/rnd:2005013 15952423

[13] Cao R Liu Y Wang Q Zhang Q Yang D Hui L. The impact of ocean acidification and cadmium on the immune responses of pacific oyster. Crassostrea gigas. Fish Shellfish Immun (2018) 81:456–62. doi: 10.1016/j.fsi.2018.07.055 30064018

[14] Sevcikova M Modra H Slaninova A Svobodova Z. Metals as a cause of oxidative stress in fish: a review. Veterinární Medicína. (2011) 56(11):537–46. doi: 10.17221/4272-VETMED

[15] Firat O Çogun HY Aslanyavrusu S Kargın F. Antioxidant responses and metal accumulation in tissues of nile tilapia *Oreochromis niloticus* under zn, cd and zn + cd exposures. J Appl Toxicol (2010) 29(4):295–301. doi: 10.1002/jat.1406 19058294

[16] Musharraf M Khan MA. Dietary manganese requirement of fingerling Indian major carp, *Labeo rohita* (Hamilton) estimated by growth, tissue manganese concentration and hepatic manganese-superoxide dismutase activity. Aquaculture (2021) 540:736734. doi: 10.1016/j.aquaculture.2021.736734

[17] Chen C Zhu W Wu F Liu M Tan Q Han D. Quantifying the dietary potassium requirement of subadult grass carp (*Ctenopharyngodon idellus*). Aquacult Nutr (2016) 22:541–9. doi: 10.1111/anu.12279

[18] Sharf Y Khan MA. Effect of dietary isoleucine level on growth, protein retention efficiency, haematological parameter, lysozyme activity and serum antioxidant status of fingerling *Channa punctatus* (Bloch). Aquacult Nutr (2020) 26(3):908–20. doi: 10.1111/anu.13049

[19] Coutinho F Castro C Rufino-Palomares E Ordonez-Grande B Gallardo M Oliva-Teles A. Dietary glutamine supplementation effects on amino acid metabolism, intestinal nutrient absorption capacity and antioxidant response of gilthead sea bream (*Sparus aurata*) juveniles. Comp Biochem Physiol A Mol Integr Physiol (2016) 191:9–17. doi: 10.1016/j.cbpa.2015.09.012 26424608

[20] Elmada CZ Huang W Jin M Liang X Mai K Zhou Q. The effect of dietary methionine on growth, antioxidant capacity, innate immune response and disease resistance of juvenile yellow catfish (*Pelteobagrus fulvidraco*). Aquacult Nutr (2016) 22:1163–73. doi: 10.1111/anu.12363

[21] Liang HL Ji K Ge XP Zhu J Ren MC Mi HF. Methionine played a positive role in improving the intestinal digestion capacity, anti-inflammatory reaction and oxidation resistance of grass carp, *Ctenopharyngodon idella*, fry. Fish Shellfish Immun (2022) 128:389–97. doi: 10.1016/J.FSI.2022.07.066 35940539

[22] Ji K Liang HL Ren MC Ge XP Liu B Xi BW. Effects of dietary tryptophan levels on antioxidant status and immunity for juvenile blunt snout bream (*Megalobrama amblycephala*) involved in Nrf2 and TOR signaling pathway. Fish Shellfish Immun (2019) 93:474–83. doi: 10.1016/j.fsi.2019.08.006 31381972

[23] Jiang WD Wen HL Liu Y Jiang J Kuang SY Wu P. The tight junction protein transcript abundance changes and oxidative damage by tryptophan deficiency or excess are related to the modulation of the signalling molecules, NF-κB p65, TOR, caspase-(3, 8, 9) and Nrf2 mRNA levels, in the gill of young grass carp (*Ctenopharyngodon idellus*). Fish Shellfish Immun (2015) 46:168–80. doi: 10.1016/j.fsi.2015.06.002 26057461

[24] Berg RD. The indigenous gastrointestinal microflora. Trends Microbiol (1996) 4(11):430–5. doi: 10.1016/0966-842X(96)10057-3 8950812

[25] Gomez D Sunyer JO Salinas I. The mucosal immune system of fish: the evolution of tolerating commensals while fighting pathogens. Fish Shellfish Immun (2013) 35(6):1729–39. doi: 10.1016/j.fsi.2013.09.032 PMC3963484 24099804

[26] Martin-Subero M Anderson G Kanchanatawan B Berk M Maes M. Comorbidity between depression and inflammatory bowel disease explained by immune-inflammatory, oxidative, and nitrosative stress; tryptophan catabolite; and gut-brain pathways. CNS Spectrums. (2015) 21(2):184–98. doi: 10.1017/S1092852915000449 26307347

[27] Sluijters DV Dubbelhuis PF Blommaart E Meijer AJ. Amino-acid-dependent signal transduction. Biochem J (2000) 351(3):545–50. doi: 10.1042/bj3510545 PMC1221392 11042107

[28] Shah OJ Anthony JC. 4e-bp1 and s6k1: Translational integration sites for nutritional and hormonal information in muscle. Am J Physiol-Endoc M. (2000) 279(4):E715–29. doi: 10.1152/ajpendo.2000.279.4.E715 11001751

[29] Gershon MD. 5-hydroxytryptamine (serotonin) in the gastrointestinal tract. Curr Opin Endocrinol (2013) 20(1):14–21. doi: 10.1097/MED.0b013e32835bc703 PMC3708472 23222853

[30] Fang H Wu C Pan L Li N Dong Z Zhu Q. Functions and signaling pathways of amino acids in intestinal inflammation. BioMed Res Int (2018) 2018:9171905. doi: 10.1155/2018/9171905 29682569 PMC5846438

[31] Ekim B Magnuson B Acosta-Jaquez HA Keller JA Feener EP Fingar DC. mTOR kinase domain phosphorylation promotes mtorc1 signaling, cell growth, and cell cycle progression. Mol Cell Biol (2011) 31(14):2787–801. doi: 10.1128/MCB.05437-11 PMC3133410 21576368

[32] Wen HL Feng L Jiang WD Liu Y Jiang J Li SH. Dietary trp modulates intestinal immune response, barrier function, antioxidant status and gene expression of TOR and Nrf2 in young grass carp (*Ctenopharyngodon idella*). Fish Shellfish Immun (2014) 40:275–87. doi: 10.1016/j.fsi.2014.07.004 25047359

[33] Saitoh T Fujita N Jang MH Uematsu S Yang BG Satoh T. Loss of the autophagy protein Atg16L1 enhances endotoxin-induced il-1beta production. Nature (2008) 456(7219):264–8. doi: 10.1038/nature07383 18849965

[34] Song ZH Tong G Xiao K Jiao LF Ke YL Hu CH. L-cysteine protects intestinal integrity, attenuates intestinal inflammation and oxidant stress, and modulates NF-κB and Nrf2 pathways in weaned piglets after LPS challenge. Innate Immun-London. (2016) 22(3):152–61. doi: 10.1177/1753425916632303 26921254

[35] Zhu Y Lin G Dai Z Zhou TJ Li TT Yuan TL. L-glutamine deprivation induces autophagy and alters the mTOR and MAPK signaling pathways in porcine intestinal epithelial cells. Amino Acids (2015) 47(10):2185–97. doi: 10.1007/s00726-014-1785-0 24997162

[36] Hou YC Chu CC Ko TL Yeh CL Yeh SL. Effects of alanyl-glutamine dipeptide on the expression of colon-inflammatory mediators during the recovery phase of colitis induced by dextran sulfate sodium. Eur J Nutr (2013) 52(3):1089–98. doi: 10.1007/s00394-012-0416-3 22847641

[37] Jia R Gu ZY He Q Du JL LP C Jeney GL. Anti-oxidative, anti-inflammatory and hepatoprotective effects of radix bupleuri extract against oxidative damage in tilapia (*Oreochromis niloticus*) *via* Nrf2 and TLRs signaling pathway. Fish Shellfish Immun (2019) 93(C):395–405. doi: 10.1016/j.fsi.2019.07.080 31374313

[38] Li SW. Biological characteristics and culture techniques of ophiocephalus argus. China Fisheries (1999) 10:28–31.

[39] China Fishery statistical yearbook. Beijing: China Agriculture Press (2022).

[40] Miao SY Zhao CZ Zhu JY Hu JT Dong XJ Sun LS. Dietary soybean meal affects intestinal homoeostasis by altering the microbiota, morphology and inflammatory cytokine gene expression in northern snakehead. Sci Rep-UK. (2018) 8(1):113. doi: 10.1038/s41598-017-18430-7 PMC5758704 29311620

[41] Sagada G Chen JM Shen BQ Huang AX Sun LH Jiang JH. Optimizing protein and lipid levels in practical diet for juvenile northern snakehead fish (*Channa argus*). Anim Nutr (2017) 3:156–63. doi: 10.1016/j.aninu.2017.03.003 PMC5941117 29767108

[42] Miao SY Chang EH Han B Zhang X Liu XR Zhou ZH. Dietary tryptophan requirement of northern snakehead, *Channa argus* (Cantor, 1842). Aquaculture (2021) 542(5):736904. doi: 10.1016/j.aquaculture.2021.736904

[43] Jobling M. A short review and critique of methodology used in fish growth and nutrition studies. J Fish Biol (2010) 23(6):685–703. doi: 10.1111/j.1095-8649.1983.tb02946.x

[44] Association of Official Analytical Chemists. Official methods of analysis of official analytical chemists international. sixteenth ed. Arlington. VA: Association of Official Analytical Chemists (1995).

[45] Rajendra W. High performance liquid chromatographic determination of amino acids in biological samples by precolumn derivatization with *O*-phthaldialdehyde. J Liq Chromatogr (2006) 10(5):941–55. doi: 10.1080/01483918708066746

[46] Sierra C Guevara J Lascurain R Perez A Agundis C Zenteno E. Sialylation is modulated through maturation in hemocytes from *Macrobrachium rosenbergii* . Comp Biochem Phys C. (2001) 130(2):179–89. doi: 10.1016/S1532-0456(01)00242-3 11574287

[47] Miao SY Hu JT Wan WL Xia SD Han B Zhou YC. Effects of graded levels of starch on the non-specific immune responses, antioxidant capacities and intestinal health in Chinese mitten crab, *Eriocheir sinensis* . Fish Shellfish Immun (2020) 104:402–9. doi: 10.1016/j.fsi.2020.06.035 32562867

[48] Andersen CL Jensen JL Ørntoft TF. Normalization of real-time quantitative reverse transcription-PCR data: A model-based variance estimation approach to identify genes suited for normalization, applied to bladder and colon cancer data sets. Cancer Res (2004) 64:5245–50. doi: 10.1158/0008-5472.CAN-04-0496 15289330

[49] Vandesompele J De PK Pattyn F Poppe B Van RN De PA. Accurate normalization of real-time quantitative RT-PCR data by geometric averaging of multiple internal control genes. Genome Biol (2002) 3(7):RESEARCH0034. doi: 10.1186/gb-2002-3-7-research0034 12184808 PMC126239

[50] Shearer KD. Experimental design, statistical analysis and modelling of dietary nutrient requirement studies for fish: A critical review. Aquacult Nutr (2000) 6(2):91–102. doi: 10.1046/j.1365-2095.2000.00134.x

[51] Li YM Tian DL Chen PP Zi BB Guo RJ Wang JJ. Effects of dietary lysine on growth performance, immune organs development and expression of immune-associated genes of broilers. China Poultry. (2019) 41(04):22–7.

[52] Kadowaki T Yasui Y Takahashi Y Kohchi C Soma G Inagawa H. Comparative immunological analysis of innate immunity activation after oral administration of wheat fermented extract to teleost fish. Anticancer Res (2009) 29:4871–7. 20032450

[53] Sharf Y Khan MA. Dietary tryptophan requirement of fingerling *Channa punctatus* (Bloch) based on growth, hematological parameters, intestinal enzymes, non-specific immune response, and antioxidant capacity. Aquaculture (2023) 562:738745. doi: 10.1016/j.aquaculture.2022.738745 36682022

[54] Yu ZH Yuan H Lu Y Pang SF. [125I]Iodomelatonin binding sites in spleens of birds and mammals. Neurosci Lett (1991) 125(2):175–8. doi: 10.1016/0304-3940(91)90021-K 1652711

[55] Carrillo-Vico A Guerrero JM Lardone PJ Reiter RJ. A review of the multiple actions of melatonin on the immune system. Endocrine (2005) 27:189–200. doi: 10.1385/ENDO:27:2:189 16217132

[56] Barber DL Westermann JEM White MG. The blood cells of the Antarctic icefish *Chaenocephalus aceratus lönnberg*: Light and electron microscopic observations. J Fish Biol (1981) 19(1):11–28. doi: 10.1111/j.1095-8649.1981.tb05807.x

[57] Morrow WJW Pulsford A. Identification of peripheral blood leucocytes of the dogfish (*Scyliorhinus canicular* l.) by electron microscopy. J Fish Biol (1980) 17(4):461–75. doi: 10.1111/j.1095-8649.1980.tb02779.x

[58] Scott AL Rogers WA. Hematological effects of prolonged sublethal hypoxia on channel catfish *Ictalurus punctatus* (Rafinesque). J Fish Dis (2010) 18(5):591–601. doi: 10.1111/j.1095-8649.1981.tb03799.x

[59] Martínez-Álvarez RM Morales AE Sanz A. Antioxidant defenses in fish: biotic and abiotic factors. Rev Fish Biol Fisher. (2005) 15(1-2):75–88. doi: 10.1007/s11160-005-7846-4

[60] Davis KB. Temperature affects physiological stress responses to acute confinement in sunshine bass (*Morone chrysops* × *Morone saxatilis*). Comp Biochem Physiol A Mol Integr Physiol (2004) 139(4):433–40. doi: 10.1016/j.cbpb.2004.09.012 15596388

[61] Mao XB Lv M Yu B He J Zheng P Yu J. The effect of dietary tryptophan levels on oxidative stress of liver induced by diquat in weaned piglets. J Anim Sci Biotechno. (2014) 5(1):49. doi: 10.1186/2049-1891-5-49 PMC4373006 25810902

[62] Rana T Bera AK Das S Bhattacharya D Bandyopadhyay S Pan D. Effect of chronic intake of arsenic-contaminated water on blood oxidative stress indices in cattle in an arsenic-affected zone. Ecotox Environ Safe. (2010) 73(6):1327–32. doi: 10.1016/j.ecoenv.2010.06.002 20655591

[63] Marjara IS Chikwati EM Valen EC Krogdahl Å Bakke AM. Transcriptional regulation of IL-17A and other inflammatory markers during the development of soybean meal-induced enteropathy in the distal intestine of Atlantic salmon (*Salmo salar* l.). Cytokine (2012) 60(1):186–96. doi: 10.1016/j.cyto.2012.05.027 22795954

[64] Platzer B Baker K Vera MP Singer K Panduro M Lexmond WS. Dendritic cell-bound IgE functions to restrain allergic inflammation at mucosal sites. Mucosal Immunol (2015) 8(3):516–32. doi: 10.1038/mi.2014.85 PMC4363306 25227985

[65] Rymuszka A Adaszek Ł. Pro- and anti-inflammatory cytokine expression in carp blood and head kidney leukocytes exposed to cyanotoxin stress–an *in vitro* study. Fish Shellfish Immunol (2012) 33(2):382–8. doi: 10.1016/j.fsi.2012.05.021 22641113

[66] Aujla SJ Chan YR Zheng M Fei M Askew DJ Pociask DA. IL-22 mediates mucosal host defense against gram-negative bacterial pneumonia. Nat Med (2008) 14:275–81. doi: 10.1038/nm1710 PMC2901867 18264110

[67] Zhao J Liu Y Jiang J Wu P Jiang WD Li SH. Effects of dietary iso-leucine on the immune response, antioxidant status and gene expression in the head kidney of juvenile jian carp (*Cyprinus carpio* var. jian). Fish Shellfish Immunol (2013) 35(2):572–80. doi: 10.1016/j.fsi.2013.05.027 23742869

[68] Kaur S Lal L Sassano A Majchrzak-Kita B Srikanth M Baker DP. Regulatory effects of mammalian target of rapamycin-activated pathways in type iand II interferon signaling. J Biol Chem (2007) 282(3):1757–68. doi: 10.1074/jbc.M607365200 17114181

[69] Karin M Lin A. NF-kappaB at the crossroads of life and death. Nat Immunol (2002) 3(3):221–7. doi: 10.1038/ni0302-221 11875461

[70] Weichhart T Säemann MD. The multiple facets of mTOR in immunity. Trends Immunol (2009) 30(5):218–26. doi: 10.1016/j.it.2009.02.002 19362054

[71] Kwang SA Gautam S Bharat BA. Nuclear factor-kappa b: from clone to clinic. Curr Mol Med (2007) 7(7):619–37. doi: 10.2174/156652407782564363 18045141

[72] Bollrath J Greten FR. IKK/NF-κB and STAT3 pathways: Central signalling hubs in inflammation-mediated tumour promotion and metastasis. EMBO Rep (2009) 10:1314–9. doi: 10.1038/embor.2009.243 PMC2799209 19893576

[73] Bonizzi G Karin M. The two NF-kB activation pathways and their role in innate and adaptive immunity. Trends Immunol (2004) 25(6):280–8. doi: 10.1016/j.it.2004.03.008 15145317

[74] Lu S Zhang H Wei X Huang X Huang R. 2-dodecyl-6-methoxycyclohexa-2, 5-diene-1,4-dione isolated from averrhoa carambola l. root ameliorates diabetic nephropathy by inhibiting the TLR4/MyD88/NF-κB pathway. Diabetes Metab Synd Ob. (2019) 12:1355–63. doi: 10.2147/DMSO.S209436 PMC6689538 31496773

[75] Wang L Yang JW Lin LT Huang J Wang XR Su XT. Acupuncture attenuates inflammation in microglia of vascular dementia rats by inhibiting miR-93-mediated TLR4/MyD88/NFκB signaling pathway. Oxid Med Cell Longev (2020) 2020:8253904. doi: 10.1155/2020/8253904 32850002 PMC7441436

